# Semi-Annual Meeting of the Æsculapian Society at Palestine

**Published:** 1852-06

**Authors:** Jas. M. Logan, Thos. D. Washburn

**Affiliations:** President; Secretary


*Semi-Annual Meeting of the Æsculapian Society held at Palestine*, April 28th, 1852.

The meeting was called to order by the President, Dr. S. M'Logan. Dr. Sam'l Thompson, Dr. Chas. Johnston, Dr. Alexander, Dr. Wynn, Dr. Norton, and Dr. Washburn appeared and answered to their names. Dr. Cullers was present by invitation.

Dr. Sam'l Thompson read a very interesting paper describing a prolapsus or retroversion of the bladder, causing much protrusion of the vaginal lining and severe strangury. A catheter was introduced with much difficulty, and afterwards injections of alum water freely used.

Dr. Washburn reported some cases of severe illness, supposed to be poisoning, in which six of one family were affected, and three proved fatal.

Dr. Oliver George having left the State, sent in his resignation which was accepted.

The following resolutions were then passed:

*Resolved*, That Dr. David Adams be expelled from this Society for failure to disprove the charges notified against him of having manufactured and vended a certain nostrum, or nostrums, for the cure of ague and fever, &c., in violation of the principles of honorable practice, and of the 13th and 14th rule of our ethics.

*Resolved*, That the Secretary give due notice to Dr. J. A. Powell that charges have been preferred against him of having violated the rules of the medical profession, and the 13th and 14th article of ethics in the manufacture and vending of a certain medicine or nostrum under the name of "Powell's Liniment," and that he be requested to attend and meet such charges at the next meeting of the Society.

*Resolved*, That the Secretary serve due notice on Dr. H. H. Hays of charges having been preferred against him for having given a certificate for Dr. Powell's liniment; the giving of such certificate being a violation of the 13th and 14th rule of our ethics, and that he be requested to attend and defend himself from the same.

The subject of the certificate given by Dr. Washburn to Dr. Powell's liniment, though given with the limitation of being only applicable to brute animals, was considered a violation of the spirit of our ethical rules; but in consideration of a promise to have said certificate withdrawn from circulation, further action on this case was postponed.

Evening Session.—Dr. Wynn reported verbally two cases, one of ptyalism, and a second of engorgement and protrusion of the tongue, after the administration of mercury.

Dr. Logan reported a case of uterine hæmorrhage.

The President appointed Dr. Chas. Johnston to deliver a public address at the next meeting of the Society.

Drs. Norton, Thompson, Wynn, Lescher, and Miller, were appointed to read essays. Drs. Logan, Thompson, Wynn, and Washburn were elected delegates to the State Medical Society for June next.

On motion, it was *Resolved*, That the minutes of this meeting be furnished to the *North Western Medical and Surgical Journal*, and that the editors be requested to publish the same, and also that copies be given the local papers for publication.

On motion, adjourned to meet at Palestine the last Wednesday in October next.

JAS. M. LOGAN, President.

Thos. D. Washburn, Secretary.

